# Using Thermography to Confirm Genotypic Variation for Drought Response in Maize

**DOI:** 10.3390/ijms20092273

**Published:** 2019-05-08

**Authors:** Raphael A. C. N. Casari, Dayane S. Paiva, Vivianny N. B. Silva, Thalita M. M. Ferreira, Manoel T. Souza, Junior, Nelson G. Oliveira, Adilson K. Kobayashi, Hugo B. C. Molinari, Thiago T. Santos, Reinaldo L. Gomide, Paulo C. Magalhães, Carlos A. F. Sousa

**Affiliations:** 1 Embrapa Agroenergia, Parque Estação Biológica (PqEB), Avenida W3 Norte (Final), Brasília 70.770-901, DF, Brazil; casari.raphael@gmail.com (R.A.C.N.C.); silva.dayanepaiva@gmail.com (D.S.P.); vivianny.silva@colaborador.embrapa.br (V.N.B.S.); thalita.massaro@colaborador.embrapa.br (T.M.M.F.); manoel.souza@embrapa.br (M.T.S.J.); dr.nelson74@hotmail.com (N.G.O.); adilson.kobayashi@embrapa.br (A.K.K.); hugo.molinari@embrapa.br (H.B.C.M.); 2 Chemistry Department, Universidade Federal de Lavras, Campus Universitário, Lavras 37.200-000, MG, Brazil; 3 Embrapa Informática, Av. Dr. André Tosello, 209 - Cidade Universitária, Campinas 13.083-886, SP, Brazil; thiago.santos@embrapa.br; 4 Embrapa Milho e Sorgo, Rod. MG 424 km 45, Zona Rural, Sete Lagoas 35.701-970, MG, Brazil; reinaldo.gomide@embrapa.br (R.L.G.); paulo.magalhaes@embrapa.br (P.C.M.); 5 Embrapa Meio-Norte, Av. Duque de Caxias 5650, Teresina 64.006-245, PI, Brazil

**Keywords:** abiotic stress, canopy temperature, plant phenotyping, thermal image, water deficit, *Zea mays*

## Abstract

The feasibility of thermography as a technique for plant screening aiming at drought-tolerance has been proven by its relationship with gas exchange, biomass, and yield. In this study, unlike most of the previous, thermography was applied for phenotyping contrasting maize genotypes whose classification for drought tolerance had already been established in the field. Our objective was to determine whether thermography-based classification would discriminate the maize genotypes in a similar way as the field selection in which just grain yield was taken into account as a criterion. We evaluated gas exchange, daily water consumption, leaf relative water content, aboveground biomass, and grain yield. Indeed, the screening of maize genotypes based on canopy temperature showed similar results to traditional methods. Nevertheless, canopy temperature only partially reflected gas exchange rates and daily water consumption in plants under drought. Part of the explanation may lie in the changes that drought had caused in plant leaves and canopy structure, altering absorption and dissipation of energy, photosynthesis, transpiration, and partitioning rates. Accordingly, although there was a negative relationship between grain yield and plant canopy temperature, it does not necessarily mean that plants whose canopies were maintained cooler under drought achieved the highest yield.

## 1. Introduction

The advancement and intensification of abiotic stresses over traditional areas of crops has increased interest in studies leading to the understanding of the effects of such stresses on plants. The foremost goal behind these studies is aimed at exploring traits capable of making genotypes increasingly tolerant with satisfactory productivity under increasing environmental pressures. In line with these new challenges, water deficit stress is one of the most intensely studied for its importance, which arises from the damage caused and its extent [1,2,3,4,5,6,7].

Maize (*Zea mays*) is a key crop for food and feed security and income generation for millions of smallholder farmers in sub-Saharan Africa, Asia, and Latin America [8]. In the USA, besides these more common uses, it has also long been used for ethanol production [9]. In Brazil, the maize production chain has been traditionally focused on food and feed. Until recently, the Brazilian biofuels program depended exclusively on sugarcane, especially for ethanol production [10]. Since 2012, due to problems related to grain commercialization and storage, the Brazilian government authorized the production of ethanol from maize in the Central-West region of the country. In addition to the drought problems already faced by maize in some regions, this new perspective of use for the grain will generate a demand for higher yielding genotypes more tolerant to drought and other stresses in order to meet the new challenges.

Maize is a crop that has been widely studied for increased drought tolerance as a result of recurrent grain yield losses related to water deficit in several traditional agricultural areas around the world [4,8,11,12,13,14]. Due to its importance for human consumption, especially in developing countries, maize was chosen as one of the crops to be studied under the Generation Challenge Programme (GCP) aiming at tolerance to environmental stress (https://www.generationcp.org/). GCP was an initiative of Consultative Group on International Agricultural Research (CGIAR), which is a global partnership that unites organizations engaged in research for a food-secured future.

The National Maize and Sorghum Research Center (CNPMS) from the Brazilian Agricultural Research Corporation (Embrapa) participated in the GCP by evaluating hundreds of maize genotypes under drought conditions in the target environment. Due to the number and size of trials spread throughout several environments and also the complexity and interconnection of the research network, grain yield was the only measurable trait. Thus, grain yield under drought was used as the only criterion for drought tolerance screening of maize genotypes in study. Genotypes with the highest yield were classified as drought-tolerant, while the lowest ones were classified as drought-sensitive. Those genotypes with grain yield between the two extremes were considered as intermediate.

There are several reports in the literature showing that, under water deficit, there is a significant negative correlation between canopy temperature and yield [15,16,17]. Many quantitative trait loci for canopy temperature have been mapped which also have pleiotropic effects for biomass and yield [18,19]. For maize, in particular, it has been found that genotypes selected as most drought tolerant in terms of yield showed lower canopy temperature, as well as higher stomatal conductance [16,20]. These results indicate that thermography may be applied to maize genetic breeding in the screening for drought tolerance.

The screening of hundreds or even thousands of genotypes in different environments is a common task in a plant breeding program. For drought, plants are cultivated under environments in which stress occurs at different growth stages, varying in both duration and intensity. Due to the increasing demand for phenotyping, which normally requires celerity, precision, reliability, reproducibility, and reduction of labor and costs, the traditional methods of plant phenotyping are limited [20,21,22]. To meet such high requirements, new and modern methods have been developed to complement or even replace the traditional ones, especially those using images that correlate with parameters of interest. Thermal imaging has been widely used in studies on characterization of plants subjected to water deprivation, whether due to drought or salinity stress. It showed a high correlation with gas exchange [15,23,24] and, to a lesser extent, to biomass accumulation and yield [15,17,20,21].

In this study, thermography was applied for phenotyping of contrasting maize genotypes whose classification for drought tolerance had already been established in previous studies carried out by Embrapa/CNPMS within the scope of the GCP, as previously mentioned. For this, the most contrasting genotypes discriminated on that occasion were chosen. For maize drought tolerance classification by thermography, it was assumed that plants whose canopies showed a lower temperature under water deficit stress are more tolerant to drought than those with higher canopy temperature. Thus, the objective of this study was to determine whether thermography-based classification for drought tolerance in maize would discriminate the genotypes in a similar way as that previous selection in which just grain yield was taken into account.

## 2. Results

### 2.1. Drought Effect on Visual Aspect, Leaf Thermal Pixels and Canopy Temperature of Maize Plants

**Figure 1:**
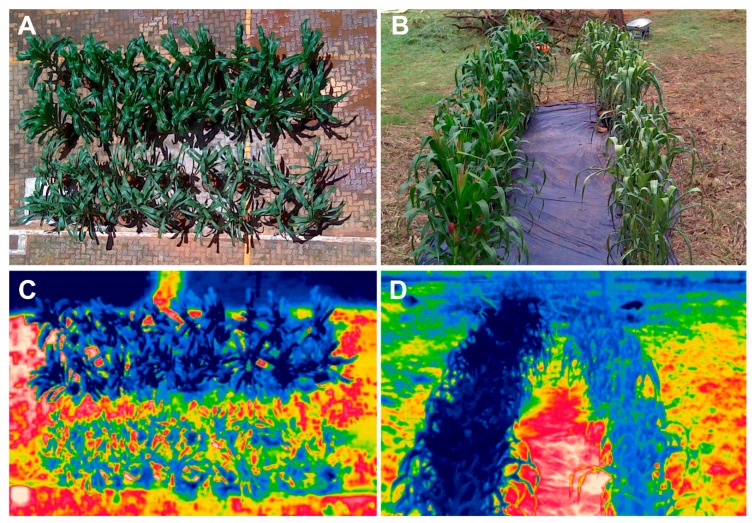
(**A**,**B**) RGB and (**C**,**D**) thermal images of maize plants taken on the 4th (**A**,**C**) and 12th (**B**,**D**) day of drought stress. The upper (**A**,**C**) and left hand side (**B**,**D**) rows of dark green (RGB) and dark blue (thermal) are controls, while the lower and right hand side rows of pale green and light blue are drought-stressed plants.

Figure 1 shows RGB and thermal images of the maize plants used in this study. Such images are composed of plants from BRS 1030, BRS 1010, 2B 707 and DKB 390 genotypes, which were selected from hundreds of phenotyped materials under field conditions. In the field phenotyping process, only the grain yield was considered as a screening criterion. Thus, the higher the grain yield the greater the tolerance, and the lower the grain yield the greater the sensitivity to drought. Based on this premise, BRS 1010 was classified as sensitive, while 2B 707 and DKB 390 were considered as drought tolerant. BRS 1030 was classified as intermediate because it showed a grain yield as low as a sensitive or as high as a tolerant, depending on the phenotyping site.

Both raw visible (Figure 1A,B) and thermal images (Figure 1C,D) taken on the 4th and 12th day of drought stress made possible visual discrimination of controls from stressed plants. Thus, controls showed green, turgid, and erect leaves, while drought-stressed plants showed characteristic symptoms, such as leaf paleness in the 4th day (Figure 1A) and subsequently, leaf wilting and bending in the 12th day (Figure 1B). False color thermal images, visualized by means of a rainbow color scale, showed drought-stressed plants as light blue, while control plants had dark blue canopies (Figure 1C,D).

**Figure 2:**
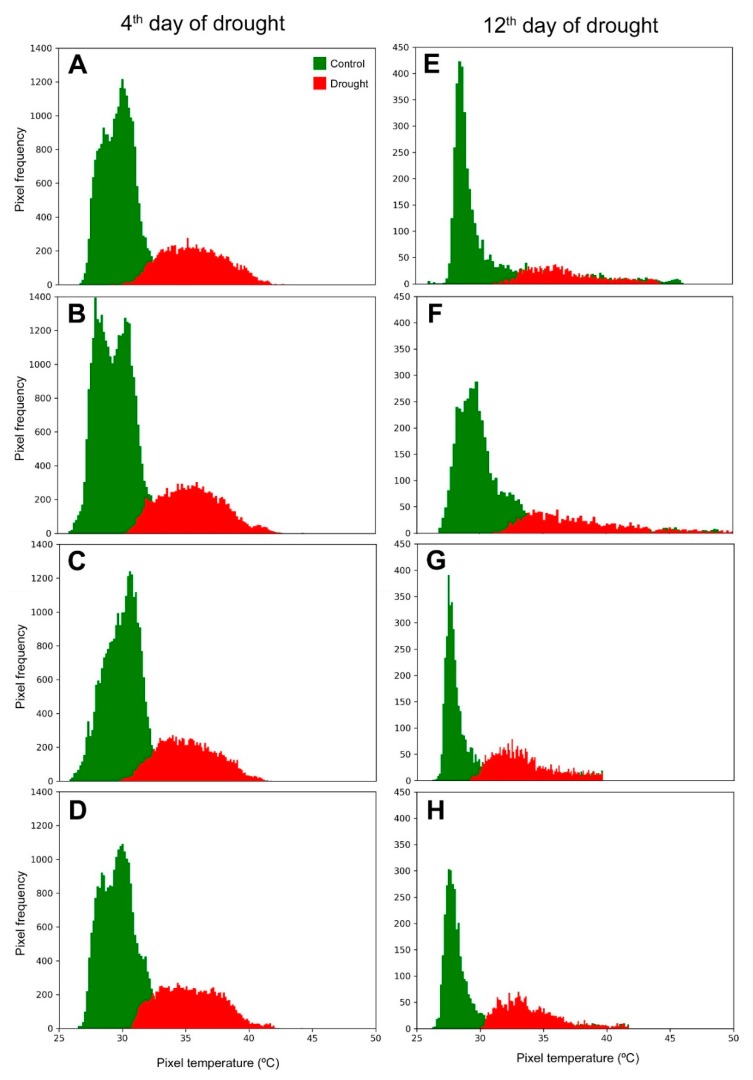
Frequency histogram of canopy pixel temperature for control and drought stressed maize plants. Panels **A**–**D** were obtained on the 4th, while **E**–**H** were obtained on the 12th day of stress. (**A**,**E**) BRS 1030; (**B**,**F**) BRS 1010; (**C**,**G**) 2B 707; (**D**,**H**) DKB 390. The pixels were segmented and extracted from thermal images taken at 15 m (**A**–**D**) and 20 m (**E**–**H**) AGL.

Based on data extracted from thermal images, frequency histograms containing temperature pixels from the 4th and 12th day of drought stress were generated (Figure 2). Despite the overlaps and the presence of outliers in both measurements, the ranges of canopy temperature were quite different from control to drought stressed plants. On average, the canopy temperatures of the drought-stressed plants were much higher than controls.

**Figure 3:**
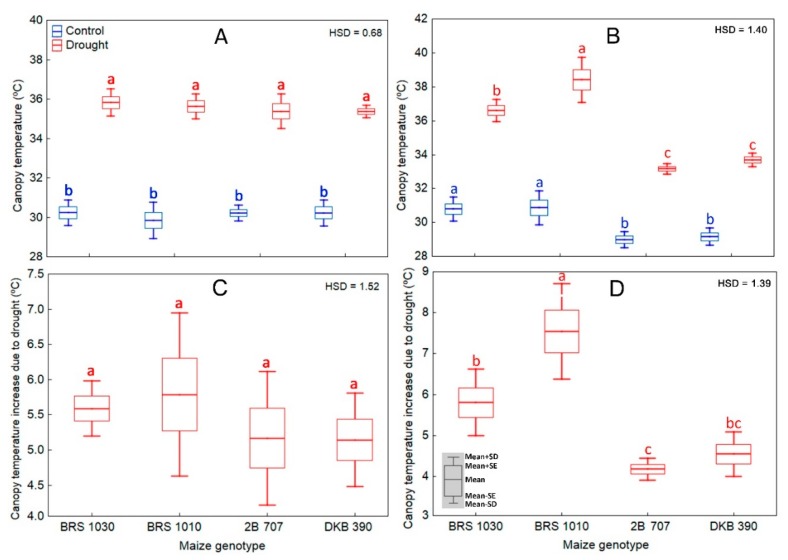
Canopy temperature for plants of maize genotypes grown in soil at field capacity (control) or subjected to water shortage (drought) at pre-flowering stage on the 4th (**A**) and 12th (**B**) day of stress. Drought-induced canopy temperature increase in maize genotypes at 4th (**C**) and 12th (**D**) day of stress. (**A**) showed significant differences between treatments regardless of genotype (*p* < 0.01), which indicates that the means of the genotypes should be compared between the treatments. Boxes from the same genotype under different treatments, topped by the same letter, are not statistically different according to Tukey’s test (*p* < 0.05). In (**B**), there was significant interaction between treatment and genotype (*p* < 0.01), which indicates that the means of the genotypes should be compared by treatment. Boxes from different genotypes under the same treatment, topped by the same letter, are not statistically different according to Tukey’s test (*p* < 0.05). In **C** and **D**, boxes from different genotypes, topped by the same letter, are not statistically different according to Tukey’s test (*p* < 0.05).

On the 4th day under stress, there were differences in canopy temperature between control and drought-stressed plants, regardless of genotype (Figure 3A). On the 12th day, there was an interaction effect between genotype and treatment for canopy temperature (Figure 3B). Thus, under control, although there was only a slight difference among them, both BRS genotypes showed higher canopy temperature than 2B 707 and DKB 390. Under water shortage, all maize plants suffered a drought-induced canopy temperature increase whose intensity was genotype-dependent. The highest canopy temperature was found in BRS 1010, followed by BRS 1030, while 2B-707 and DKB-390, which did not differ from each other, showed the lowest.

For this type of study, the plant canopy temperature itself has only minor importance. What really matters is the magnitude of drought-induced canopy temperature increase based on control plants. In this study, there was no difference on the 4th day of stress (Figure 3C). However, on the 12th day such increase was higher in BRS 1010, which was followed by BRS 1030, and the smaller occurred in 2B 707. However, BRS 1030 was not statistically different from DKB 390 which, in turn, was also not different from 2B 707 (Figure 3D).

### 2.2. Restriction on Water Supply in the Soil and Its Effects on Soil and Leaf Water Content, Plant Water Consumption, Canopy Temperature, and Gas Exchange

**Figure 4:**
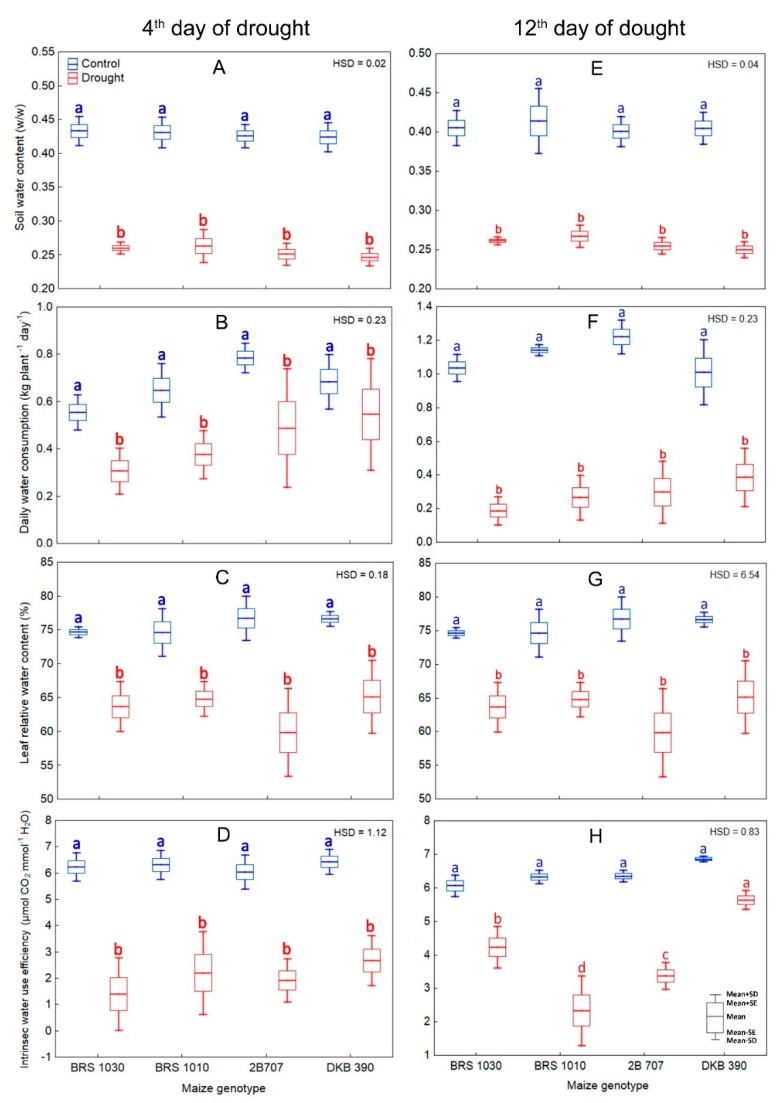
Water parameters for control and drought stressed maize plants in the 4th and 12th day of stress. (**A**,**E**) Soil water content, (**B**,**F**) leaf relative water content, (**C**,**G**) daily water consumption and (**D**,**H**) intrinsic water use efficiency in plants of maize genotypes grown in soil at field capacity (control) or subjected to water shortage (drought) at pre-flowering stage. 4th day: (**A**–**D**) showed significant differences among treatments regardless of genotype (*p* < 0.01) which indicates that the means of the genotypes should be compared between the treatments. Boxes from the same genotype under different treatments, topped by the same letter, are not statistically different according to Tukey’s test (*p* < 0.05). 12th day: (**E**–**G**) showed significant differences among treatments regardless of genotype (*p* < 0.01) which indicates that the means of the genotypes should be compared between the treatments. Boxes from the same genotype under different treatments, topped by the same letter, are not statistically different according to Tukey’s test (*p* < 0.05). (**H**) showed significant interaction between treatment and genotype (*p* < 0.01) which indicates that the means of the genotypes should be compared by treatment. Boxes from different genotypes under the same treatment, topped by the same letter, are not statistically different according to Tukey’s test (*p* < 0.05).

Water restriction imposed on maize plants led to a drop in soil and leaf water content, daily whole plant water consumption and intrinsic water use efficiency in both 4th and 12th (Figure 4) day of stress.

**Figure 5:**
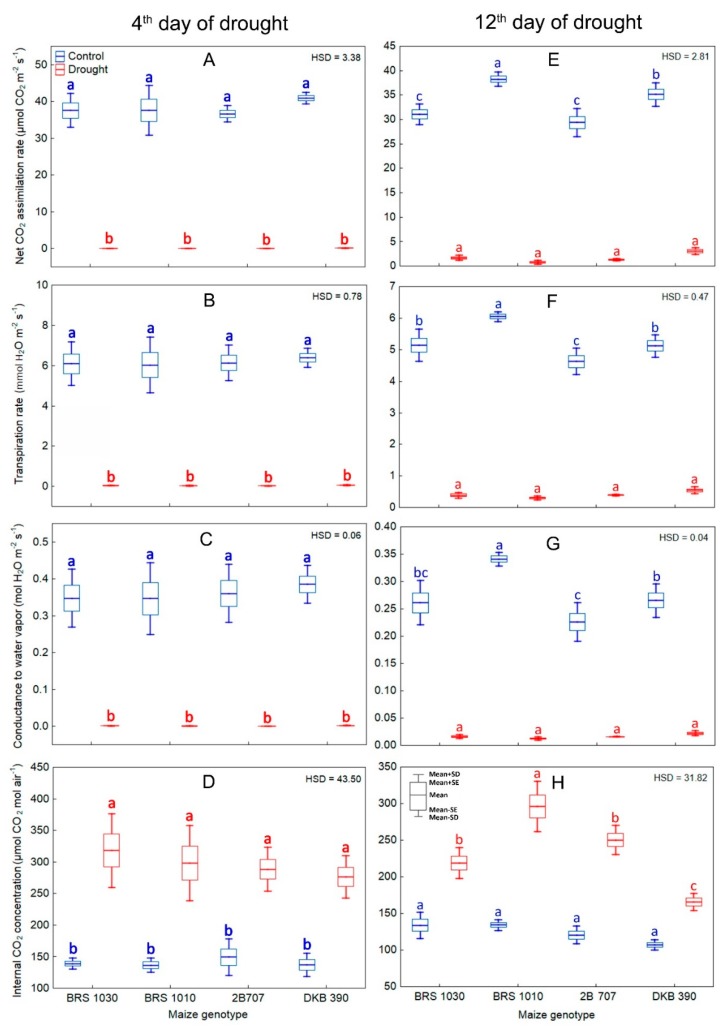
Gas exchange measurements for control and drought stressed maize plants in the 4th and 12th day of stress. (**A**,**E**) Net CO_2_ assimilation rate, (**B**,**F**) Conductance to water vapor, (**C**,**G**) Transpiration rate, (**D**,**H**) Internal CO_2_ concentration in plants of maize genotypes grown in soil at field capacity (control) or subjected to water shortage (drought) at pre-flowering stage. 4th day: (**A**–**D**) showed significant differences among treatments regardless of genotype (*p* < 0.01) which indicates that the means of the genotypes should be compared between the treatments. Boxes from the same genotype under different treatments, topped by the same letter, are not statistically different according to Tukey’s test (*p* < 0.05). 12th day: (**A**–**D**) showed significant interaction between treatment and genotype (*p* < 0.01) which indicates that the means of the genotypes should be compared by treatment. Boxes from different genotypes under the same treatment, topped by the same letter, are not statistically different according to Tukey’s test (*p* < 0.05).

In parallel, a strong reduction in leaf gas exchange occurred (Figure 5A–C,E–G). Furthermore, drought caused an increase in internal CO_2_ concentration (Ci) in all genotypes (Figure 5D,H). Differences in gas exchange rates between control and drought stressed plants were generally considerably higher than those in canopy temperature. The severity at which these characteristics were affected by drought was not genotype-dependent in the 4th day. However, in the 12th day of stress all the parameters derived from gas exchange showed interaction between treatment and genotype (Figure 4H, Figure 5E–H).

### 2.3. Effect of Drought on Biomass, Grain Yield and Main Yield-Correlated Variables

**Figure 6:**
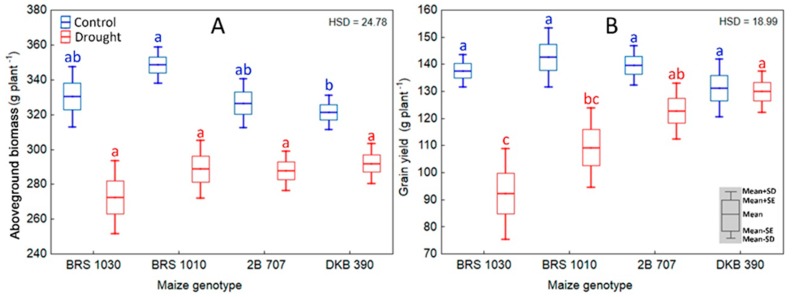
Aboveground biomass and grain yield for control and drought stressed maize plants. (**A**) Aboveground biomass and (**B**) grain yield in plants of maize genotypes grown in soil at field capacity (control) or subjected to water shortage (drought) at pre-flowering stage. **A** and **B** showed significant interaction between treatment and genotype (*p* < 0.01) which indicates that the means of the genotypes should be compared by treatment. Boxes from different genotypes under the same treatment, topped by the same letter, are not statistically different according to Tukey’s test (*p* < 0.05).

Under control conditions, the maize genotype BRS 1010 presented higher aboveground biomass than DKB 390, while the others did not differ among them. Under drought, the genotypes showed no difference in aboveground biomass (Figure 6A). Regarding grain yield, it did not differ among maize genotypes under control. However, it was strongly reduced at a genotype-dependent intensity level under drought (Figure 6B). The highest reduction in grain yield was noticed in BRS 1030 (33%), followed by BRS 1010 (23%), and the lowest occurred in 2B 707 (12%). No statistically significant reduction in grain yield was observed in DKB 390 due to drought.

**Figure 7:**
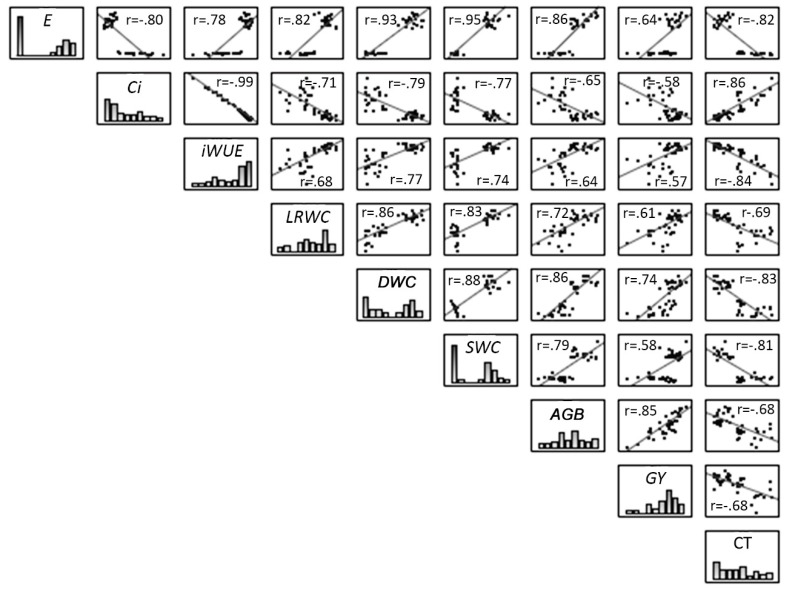
Relationship between variables measured in the experiment for control and drought stressed maize plants in the 12th day of stress, which are represented using regression lines in the plots. The numerical figures displayed provide the correlation coefficients. The *p* values was <0.01 for all the plotted variables. E – Transpiration rate, Ci – Intercellular CO_2_ concentration, iWUE – Intrinsic water use efficiency; LRWC – Leaf relative water content; DWC – Daily water consumption (water lost by evapotranspiration); SWC – Soil water content; AGB – Aboveground biomass; GY – Grain yield; CT – Canopy temperature.

At the 12th day of stress, soil and leaf water content, daily water consumption, and gas exchange measurements (represented by E), except intercellular CO_2_ concentration, showed a high positive relationship to each other, but a high negative relationship to plant canopy temperature (Figure 7). Those same variables correlated positively with aboveground biomass and grain yield, mostly in a high or moderate way. Intercellular CO_2_ concentration, in turn, correlated positively with canopy temperature, but negatively with the other variables. The relationship of canopy temperature to aboveground biomass and grain yield was negative and considered as moderate.

### 2.4. Water Deficit Stress and Its Effects on Maize Plant Leaf and Canopy Architecture

**Figure 8:**
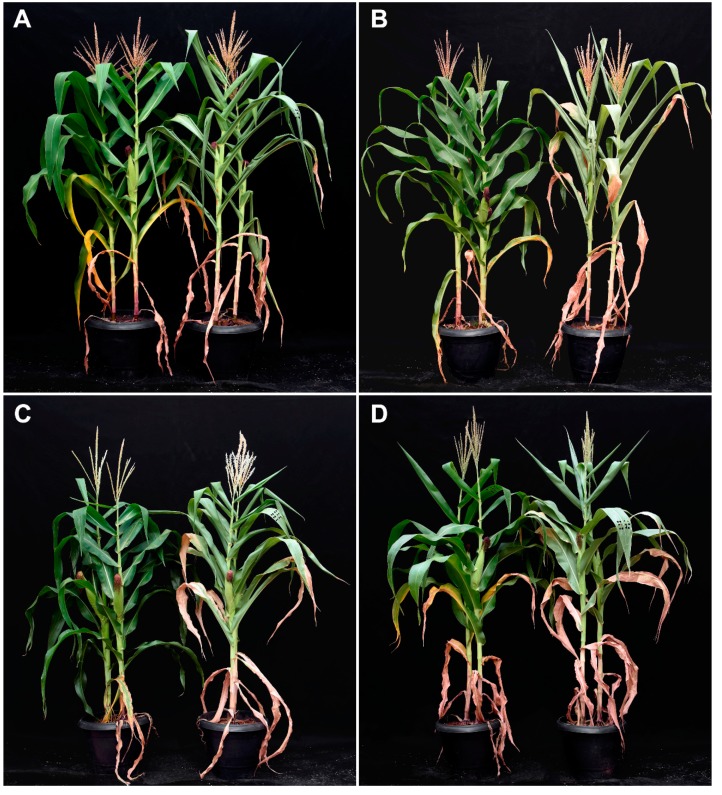
Drought effects on leaf characteristics and canopy architecture of maize plants. (**A**) BRS 1030, (**B**) BRS 1010, (**C**) 2B 707, (**D**) DKB 390. Plants on the left hand side were grown in soil at field capacity (control), while those on the right hand side were subjected to soil water shortage (drought) for 12 consecutive days at pre-flowering stage. Images were taken on the 12th day under drought.

**Figure 9:**
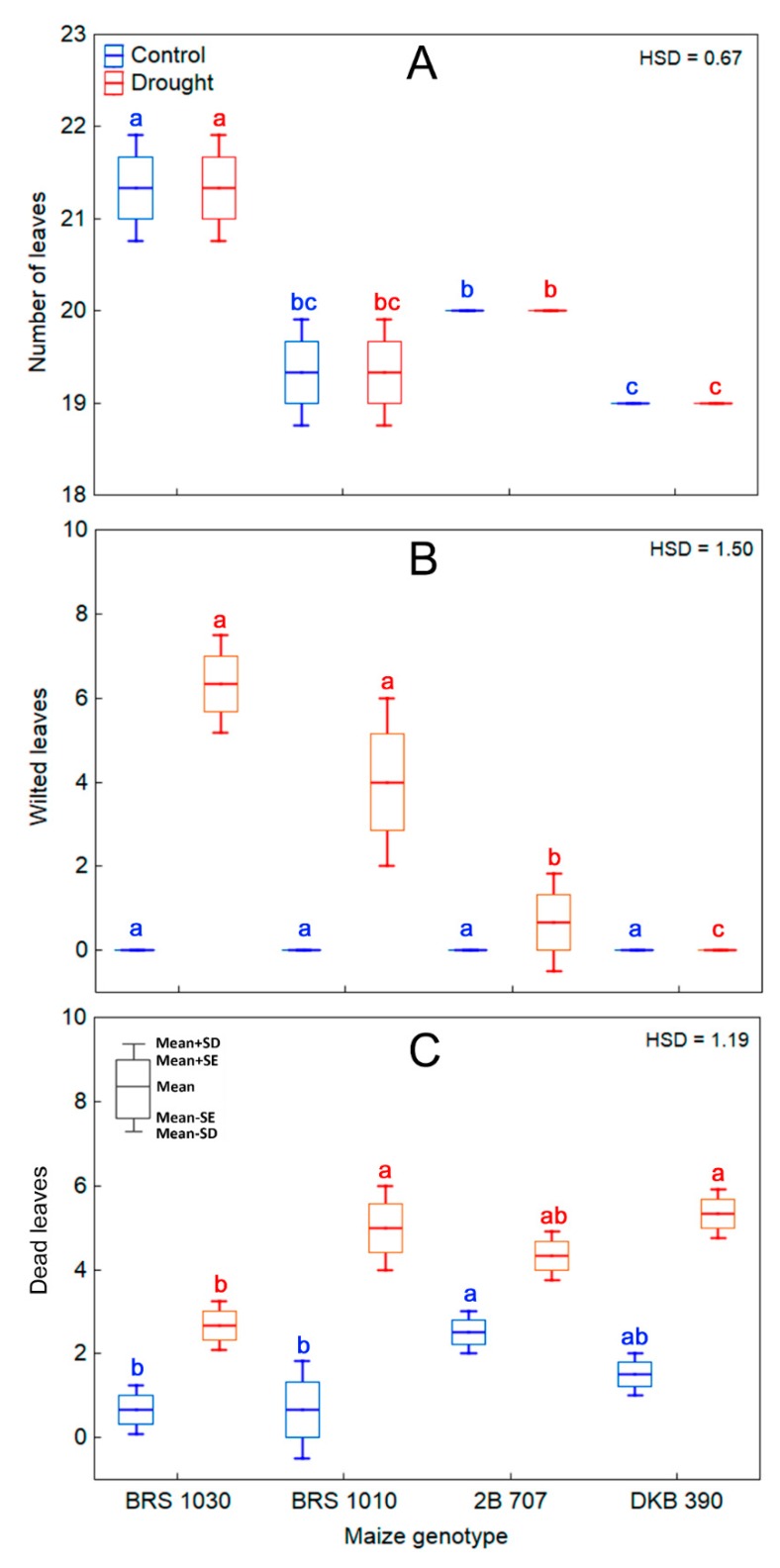
Drought effects on total, wilted and dead leaves of maize genotypes. (**A**) Number of total (**B**) wilted and (**C**) dead leaves in plants of maize genotypes grown in soil at field capacity (control) or subjected to water shortage (drought) at pre-flowering stage. (**A**–**C**) showed significant interaction between treatment and genotype (*p* < 0.05) which indicates that the means of the genotypes should be compared by treatment. Boxes from different genotypes under the same treatment, topped by the same letter, are not statistically different according to Tukey’s test (*p* < 0.05).

**Figure 10:**
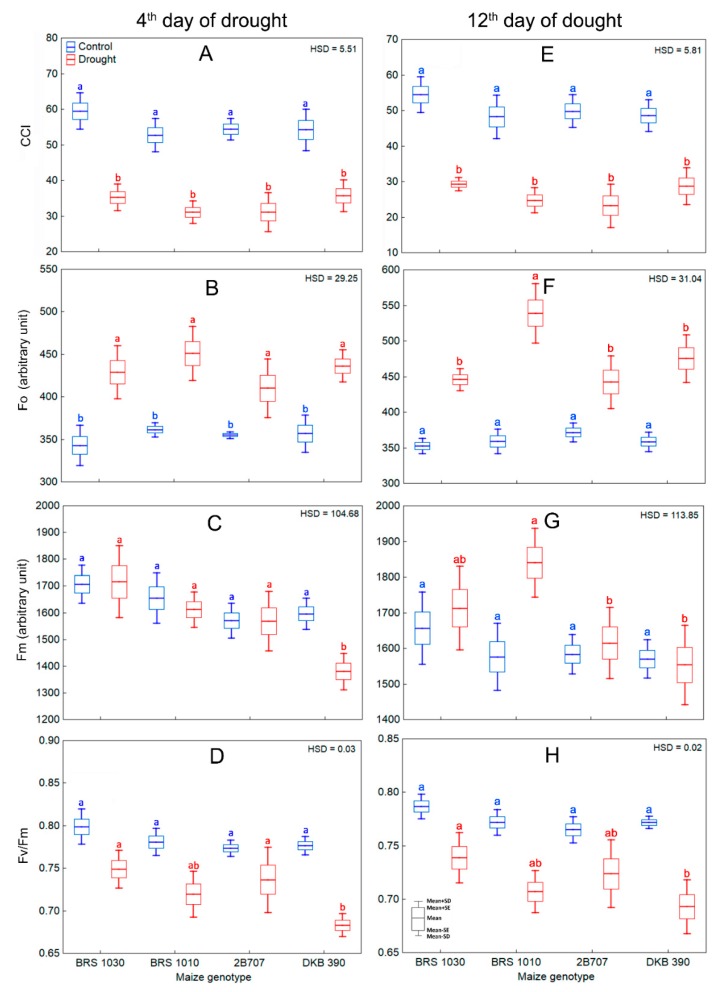
Chlorophyll content and variables derived from chlorophyll fluorescence measurements for control and drought stressed maize plants in the 4th and 12th day of stress. (**A**,**E**) Chlorophyll content index (CCI), (**B**,**F**) minimum fluorescence yield on dark-adapted leaf (Fo), (**C**,**G**) maximum fluorescence yield on dark-adapted leaf (Fm), (**D**,**H**) maximum photosystem II quantum yield (Fv/Fm) in plants of maize genotypes grown in soil at field capacity (control) or subjected to water shortage (drought) at pre-flowering stage. 4th day: (**A**,**B**) showed significant differences among treatments regardless of genotype (*p* < 0.01), which indicates that the means of the genotypes should be compared between the treatments. Boxes from the same genotype under different treatments, topped by the same letter, are not statistically different according to Tukey’s test (*p* < 0.05); **C**–**D** showed significant interaction between treatment and genotype (*p* < 0.05) which indicates that the means of the genotypes should be compared by treatment. Boxes from different genotypes under the same treatment, topped by the same letter, are not statistically different according to Tukey’s test (*p* < 0.05). 12th day: (**E**) showed significant differences among treatments regardless of genotype (*p* < 0.01), which indicates that the means of the genotypes should be compared between the treatments. Boxes from the same genotype under different treatments, topped by the same letter, are not statistically different according to Tukey’s test (*p* < 0.05); (**F**–**H**) showed significant interaction between treatment and genotype (*p* < 0.01) which indicates that the means of the genotypes should be compared by treatment. Boxes from different genotypes under the same treatment, topped by the same letter, are not statistically different according to Tukey’s test (*p* < 0.05).

Maize control plants maintained green, turgid, and erected leaves, keeping canopy morphological characteristics typical of plants grown under non-stressing conditions (Figure 8A–D; Figure 9A–C). In agreement with the visual observations, these plants had leaves with high values of chlorophyll content index and their photochemical variables clearly differed from those of the stressed plants (Figure 10A–H). However, drought at the pre-flowering stage affected leaves and canopy architecture in an intensity depending on the genotype. Such effects visually seemed less drastic for BRS 1030 which most of the leaves were greener than other genotypes, although they were rolled, wilted and bent (Figure 8A and Figure 9B). For BRS 1010, drought caused an acceleration of leaf senescence process of the canopy (Figure 8B and Figure 9C). For both 2B 707 (Figure 8C) and DKB 390 (Figure 8D), practically no wilting or leaf rolling occurred (Figure 9B). For these genotypes, leaf senescence was just noticed in the lower older leaves, while leaf paleness was its hallmark under drought.

Clearly, there was chlorophyll breakdown (Figure 10A,E) since the leaves were pale, which was gradually manifested along the experiment. In addition, there was an increase in the minimum fluorescence yield on dark-adapted leaf (Fo) for all maize genotypes under drought, especially in BRS 1010 at the 12th day of stress (Figure 10B,F). Although drought has not caused much changes in the maximum fluorescence yield on dark-adapted leaf (Fm), it was smaller in DKB 390 at the 4th (Figure 10C) and in both 2B 707 and DKB 390 at the 12th (Figure 10G) day of stress. The consequence was a reduction in the maximum photosystem II quantum yield (Fv/Fm), but just for DKB 390 in relation to BRS 1030 e 2B 707 at the 4th day (Figure 10D) and in relation to BRS 1030 at the 12th day (Figure 10H).

## 3. Discussion

### 3.1. Relationship Among Canopy Temperature, Gas Exchange, Leaf Water Content, Water Consumption, and Yield for Maize Plants under Water Restriction

The first line of defense of plants against water stress is stomatal closure, with a consequent decrease in water vapor and CO_2_ conductance [25,26]. In fact, concomitantly with an increase in canopy temperature (Figure 1, Figure 2 and Figure 3), there was a strong and proportional reduction in water parameters and gas exchange rates both at the beginning (4th day; Figure 4A–D; Figure 5A–C) and at the end (12th day; Figure 4E,F and Figure 5E,F) of the stress. In both time periods of drought, the discrimination between control and stressed plants could be performed by canopy temperature. However, only at the end of the experiment could the genotypes submitted to drought be discriminated from each other based on that trait (Figure 3B,D).

On the 4th day, the leaves of the stressed plants presented only a slight wilt and paleness (Figure 1A), but there was no difference in canopy temperature between them (Figure 3C). Canopy temperature difference among stressed plant genotypes only occurred on the 12th day (Figure 3D), when there were already signs that additional mechanisms to cope with the stressful condition were manifesting. Most likely, the regulation of plant canopy temperature depended almost totally on stomata at the onset of stress, while at the end of stress other physiological mechanisms were triggered. Such mechanisms work in all the levels of organization [27]. At the organ level, we visually observed leaf senescence, wilting, and rolling, as well as leaf angle changes.

Although we are dealing with measurements taken on different scales (Figure 3, Figure 4, Figure 5 and Figure 6), on a small scale, leaf relative water content (Figure 4B,F) and gas exchange (Figure 5A–C,E–G) were obtained in the more metabolically active leaves. We assumed that they represent the plant as a whole. Thus, being an isohydric species, maize close their stomata in response to a decrease in soil water and/or an increase in vapor pressure deficit to control plant water potential [28]. Accordingly, stomatal conductance has been considered as a better indicator of soil moisture than water potential under drought conditions [29]. Since stomatal closure raises canopy temperature, drought tolerance screening based on thermal imaging can be suited to maize. However, some authors [30,31] have shown that the relationship between stomatal conductance and leaf temperature (or derived parameters), in spite of the difference in scales, is not always as strong as that obtained in a highly controlled environment [15]. This means that the scale of the measurements may even have some influence, but it is probably not a determinant factor. In our study, whole plant water consumption (Figure 4C,G) or leaf gas exchange (Figure 5A–C,E–G) were not different among maize genotypes under drought, although there were differences in canopy temperature among them, but only in the 12th day. Furthermore, neither daily water consumption nor gas exchange individually were highly correlated to the increase in canopy temperature of maize genotypes as a function of drought (Figure 7). This implies that in addition to those already mentioned, other traits that help regulate leaf temperature in plants as a whole may be acting under a prolonged drought.

It should be kept in mind that, by the energy balance equation, the two most important variables for leaf temperature regulation are absorbed radiation and stomatal conductance [28,29]. The former adds energy and heats the leaf [30,32]; the latter acts to cool it down [15,23,28,29]. Leaf heat loss occurs mainly due to stomatal transpiration [15,23,28,29]. Therefore, stomatal conductance accounts for most of the change in canopy temperature [20]. However, besides stomatal conductance and transpiration, there are additional mechanisms involved in leaf temperature regulation [32,33]. Instead of leaf cooling, such mechanisms probably avoid leaf heating [34,35,36,37,38].

Both transpiration cooling and avoidance of excessive leaf heating strategies work to regulate canopy temperature. The former has been usually related to yield, while the latter is primarily aimed at survival. It turned out that the maize genotypes that had the highest grain yield under drought (Figure 6B) were those that also showed lower canopy temperature increase in the 12th day of stress (Figure 3D). As under drought, the levels of daily water consumption (Figure 4C,G) or gas exchange rates (Figure 5A–C,E–G) remained remarkably low and there was practically no difference among them, it was assumed that the cooling rate due to plant water loss was similar for all. Therefore, it is likely that maize genotypes showing lower canopy temperatures under drought benefited from avoidance of excessive leaf heating in a correlated way to grain yield (Figure 7).

### 3.2. Implications of Water Deficit Stress in the Plant Leaves and Canopy Architecture

Under drought, BRS 1030 showed delayed leaf senescence, leaf wilting, and rolling, which were associated with a change in leaf angle (Figure 8A and Figure 9B,C). These last traits have been cited in the literature as strategies to reduce light energy absorption and preventing canopy overheating [36,37]. On the other hand, BRS 1010 showed a senescence acceleration in most of the leaves (Figure 8B; Figure 9C; Figure 10F), leading to dysfunction of the stomata and, consequently, loss or severe restriction of the main mechanism responsible for leaf cooling. We suppose that such distinct and contrasting strategies worked for both genotypes, but they led to an increase in canopy temperature and represented additional costs, which resulted in a drop in the yield at different intensities. BRS 1030 probably spent a lot of energy to keep your leaves alive, while BRS 1010 early lost part of its photosynthetically active leaf surface.

The results obtained in this study corroborate the statement that there are several traits recognized for conferring drought tolerance in plants, but the performance of each of them depends on the scenario [2]. According to [3], the strategy used by genotypes like BRS 1030 can be adequate when grown in soils with appreciable water reserves so that their roots could explore as they extend through the soil profile. It is also useful for plants grown in regions subject to short drought period under vegetative phase, in which plant growth could rapidly be resumed after a rainfall, since its leaves are kept green. However, in this study, since the roots were confined in pots under water limitation, such a strategy was not effective. Most of the leaves of 2B 707 and DKB 390 become very pale. Most likely, this strategy reduced light energy absorption, avoiding excessive canopy heating. Probably, by mainly reducing the synthesis of photosynthetic pigments, they were able to maintain a positive carbon balance, which could be directed to obtain higher grain yields.

For the before-mentioned reasons, it does not necessarily mean that the coldest genotypes will be the highest yielding and the hottest, the least. In this type of study, the correlation between temperature and biomass has been much lower than for gas exchange [15] and even lower for yield [17,20]). This implies that for more integrative characteristics, additional factors are involved, such as respiration rate [39] and partitioning of assimilates [40]. Even so, based on the results obtained in this study, we can state that thermography can be considered as a potential tool for selecting drought-tolerant plants in the early stages of a plant-breeding program. The technique is capable of selecting plants whose canopies heat less, which can lead to a higher yield under drought stress. However, one should be aware of the physiological responses of the plants to drought in order to choose the right moment to assess the canopy temperature. This is important because most of the mechanisms involved in regulating canopy temperature should be active at the time of canopy temperature assessment.

## 4. Materials and Methods

### 4.1. Plant Material and Growth Conditions

The experiments were carried out in a greenhouse at the National Agroenergy Research Center (https://www.embrapa.br/en/agroenergia), in Brasilia, Brazil (S—15.732°, W—47.900°) from December 8, 2016 (sowing) to May 2, 2017 (harvesting). Light intensity, temperature, and air relative humidity fluctuated accordingly to environment. Such weather variables were monitored and recorded during the most important maize plant growth stages (Supplementary File 1).

We used four maize genotypes showing contrasting drought responses (BRS1010, BRS-1030, 2B-707, and DKB-390). Such genotypes had previously been screened based on results obtained in the target environment in a 5-year series of field experiments carried out by Embrapa/CNPMS. Thus, based purely on grain yield, they were classified as sensitive, intermediate, and tolerant. BRS-1010 was classified as sensitive because it showed the lowest yield. 2B-707 and DKB 390 were considered as tolerant because they showed the highest yield. BRS 1030 was considered intermediate because it showed yield as high as a tolerant or as low as a sensitive, depending on the phenotyping site [41,42]. Seeds were sown in pots (20 kg) filled with typical soil used for maize growing in Brazil (dystrophic Red Latosol according to Brazilian Soil Classification). Based on some soil physical–chemical analyses, lime and fertilizer were added to provide optimal plant growth. Two maize plants were held per pot. They were grown on a daily replenishment of water at field capacity (100% of the available water).

### 4.2. Measured Variables

#### 4.2.1. Soil and Leaf Water Content for Control and Drought Stress Maize Plants

Water supply to the soil was rigorously managed throughout the experiment for both control and drought stressed plants. Soil moisture was daily returned to the level according to the water regime to which they were submitted. For drought-stressed plants, water supply was withheld at 54 days after sowing (1 February 2017), when they reached V12 growth stage (12th leaf collar visible). The plants became actually stressed two days later (3 February 2017), when gas-exchange rates approached zero. The drought stress level was maintained for 12 consecutive days (until 14 February 2017), while another group of plants remained with water replenished to field capacity (control).

Determination of soil water holding capacity was performed by watering the pots with excess water until saturation. Then, the excess of water was naturally allowed to drain, avoiding evaporation. Lastly, the weight of the soil with the maximum amount of water it could retain was obtained. This resulting weight was considered as the soil weight at field capacity. Based on this weight, the control plants were daily watered for replenishment of the evapotranspired water. The plants under drought stress had water withheld until the gas exchange approached zero. Then, the soil water content was determined and maintained at this level. Soil water content (SWC) was measured based on the gravimetric method. Soil sample was oven dried at 105 °C until constant weight. The daily water consumption (DWC) by evapotranspiration and the remaining soil water content (SWC) was recorded for both control and drought stressed plants. The amount of water lost was replenished daily to return to the water levels according to the treatment to which the plants were subjected.

For leaf relative water content (LRWC), a leaf piece was removed from the same portion of a leaf inserted in the same position of the canopy as those used for gas exchange measurements, but in another plant. Since each pot contained two plants, which were sown at the same time and, therefore, were at the same age and growth stage, they experienced the same soil water content. Accordingly, we assumed that their correspondent leaves had similar relative water content. The fresh weight (FW) of the leaf piece was immediately determined after removal. The leaf piece was then immersed on distilled water in darkness at 4 °C/24 h before determination of turgid weight (TW). It was then placed into a paper envelope and dried at 65 °C/24 h to determine dry weight (DW). RWC was calculated as in [43] by the following equation: $LRWC=\frac{\left(FW-DW\right)}{\left(TW-DW\right)}\times 100$

#### 4.2.2. Schedule for Gas Exchange and Thermal Imaging Measurement

Both thermal and gas exchange measurements were taken on the 4th and 12th day of drought stress nearly simultaneously close to midday. Around this time, the sun is typically close to the zenith position in the tropics. Under such conditions, transpiration is high for maize, as a C4 plant, which helps to enhance differences among genotypes [20,44] and different water regimes [45].

#### 4.2.3. Gas Exchange Measurements

Gas exchange measurements were conducted on the middle third of the healthy and fully expanded leaf 12, in a previously marked area. A LI-COR mod. 6400XT (LI-COR, Lincoln, NE, USA) infrared gas analyzer, equipped with a size measuring head with 2 × 3 cm and an artificial LED lighting system model 6400-02B, was used. It was set up to hold relative humidity between 50–60% and light intensity of 2000 μmol m^−2^ s^−1^ in the measuring head, block temperature at 30 °C, and flow rate at 500 μmol s^−1^. CO_2_ concentration on the reference cell was controlled at 400 ppm using a CO_2_ mixer model 6400-01. The following gas exchange variables were evaluated: net CO_2_ assimilation rate (A), conductance to water vapor (*gs*), transpiration rate (*E*) and intercellular CO_2_ concentration (*Ci*). They were calculated based on equations described in the LI-COR 6400XT user manual (www.licor.com/documents/s8zyqu2vwndny903qutg). A measure of transpiration efficiency at the leaf level was determined as intrinsic water use efficiency (*iWUE*). It was calculated as the ratio of instantaneous net CO_2_ assimilation (*A*) to transpiration (*E*) = *A*/*E* [46].

#### 4.2.4. Chlorophyll Content Index and Chlorophyll Fluorescence Measures

Both measures were performed on leaf 12, in the same portion used for gas exchange and relative water content measures. Chlorophyll content index (CCI) was analyzed using a chlorophyll meter Opti-science model CCM-200 Plus (Opti-Sciences Inc., Hudson, NH, USA). Measures were taken in five different points of the selected leaf portion. The averaged values were used. For chlorophyll fluorescence measurements, the maize plants were analyzed by means of the chlorophyll fluorescence technique, using a portable fluorometer Walz Mod. Mini-PAM. (Heinz Walz GmbH, Effeltrich, Bavaria, Germany). The device was configured to provide an initial fluorescence measurement light with frequency of 0.6 Hz and intensity at level 2, with amplifier factor of the detector’s electronic signal set up at level 1. The intensity of the saturating light pulse was set up at level 12. The piece of the leaf to be evaluated was previously kept in the dark for at least 1 h with the aid of a leaf clip Heinz Walz Mod. DLC-8. For the measurement procedure, the end of the fluorescence probe was coupled to the leaf clip, which was attached to the leaf. Then, the leaf clip shutter was opened to allow exposure of the leaf piece to be measured. After 2 s, enough time for the initial fluorescence signal (without influence of actinic light) to stabilize, the “start” button was pressed, triggering the maximum photosystem II quantum yield (*Fv*/*Fm*) by using the WinControl software. Initially, a routine recorded the minimum fluorescence yield on dark-adapted leaf (Fo), generated by the initial measurement light. Then, the software triggered a pulse of saturating light and recorded the maximum fluorescence yield on dark-adapted leaf (Fm). From these two variables, the internal algorithm present in the WinControl software automatically calculated Fv/Fm, according to the equation: $\frac{Fv}{Fm}=\frac{\left(Fm-Fo\right)}{Fm}$.

#### 4.2.5. Total, Wilted, and Dead Leaves

Leaf emission was recorded throughout the cycle of maize plants. At the end of the experiment, we counted the wilted and dead leaves due to the drought. The wilted leaves were visually identified. The dead leaves were those in which more than 50% of the leaf blade area was senescent.

#### 4.2.6. Thermal Image Capturing and Processing

The thermal images on 4th day were taken manually, while those on the 12th day were taken from an unmanned aerial vehicle (UAV). The former were taken at a height of 15 m from the canopy, while the latter at 20 m. Therefore, each pixel covered an area of, at least, 2.8 × 2.8 cm. A pixel with such size is suitable for an adult maize plant as it is less than the average width of a single leaf. Furthermore, it allows an acceptable spatial resolution and accurate temperature measurement of the leaves.

An UAV XFly Mod. X800 (XFly Brasil, Bauru, SP, Brazil) was used to carry the thermal imager (Supplementary File 2). It was equipped with a Pixhawk flight controller, which is able to make GPS waypoint navigation and altitude control. Its payload was up to 3 kg and was able to fly in autonomous or manual mode. It had a flying autonomy of around 25 min when carrying the thermal imager. Both UAV and thermal imager were controlled from a ground station. The former using a free open-source software known as Mission Planner. The latter using a Flir proprietary software remotely operated. A carbon fiber gimbal was specially developed for attachment of the thermal imager to the UAV. This solution allowed remote control, positioning, focusing, and triggering of the camera on the target. Captured images could be transmitted in real time to the monitoring ground station. Both thermal and visible images were obtained by using a Flir camera mod. T-420 (Flir Systems, Danderyd, Sweden) which had the following characteristics: 320 × 240 pixel of thermal spatial resolution, spectral response from 7.5 to 13 µm, uncooled microbolometer focal-plane array detector, 60 Hz of frame rate, pixel thermal sensitivity of 0.045 °C at 30 °C, temperature accuracy of ±2 °C or 2% of reading (whichever is greater) at 25 °C, equipped with a lens of 25°, and weight of 0.88 kg including battery. Following the principles of infrared thermography [47], the thermal imager was set up for the environmental conditions at the time of image capture, which included distance of the plant canopies, reflected temperature (aluminum foil method), atmospheric temperature, and air relative humidity. Canopy emissivity was set to 0.98. For this purpose, the plant canopies were considered as greybodies in which emissivity is constant and independent of the wavelength for the spectral range of 7.5–13 µm.

The images used for calculating canopy temperature were captured with the smallest normal view angle to the ground to avoid any temperature variation due to angular effect [48]. For image capture, the pots containing maize plants were removed from the greenhouse. Then, they were placed on the ground and plants were left to acclimate to the external environmental conditions for a few hours.

The poor accuracy of the temperature measurement of thermal cameras, including the one we used in this work, has not been an issue in this type of study. This is because what really matters is the canopy temperature difference between control and stressed plants and not the accurate canopy temperature. To make sure we were measuring the most precise temperature difference among the plant canopies, we compared several images taken at the same distance and angle to check the repeatability (precision) of the canopy temperature measurement, which was high. In addition, thermal images were taken in a short time interval, which was less than five minutes, to ensure that genetic factors, instead of weather variables changes, would be responsible for temperature differences among plant canopies. Accordingly, there was no need to use any thermal index or mathematical artifices for data normalizing in order to compare them. Thus, we assumed that differences in mean canopy temperature between control and drought stressed plants would only reflect traits expressed by the genotypes. Thermal images were saved in a raw format, as a non-compressed array, while RGB images were saved as JPEG files.

For temperature distribution in the maize plant canopies, RGB images obtained by the visible sensor of the thermal camera were employed to identify plant leaf pixels in the thermogram. The procedure consisted of some steps, which are segmentation, alignment, and masking. In this study, we applied color segmentation. Thus, color images were converted from the original RGB color space to the CIELAB space [49] and just the color channels a* and b* in CIELAB were employed in the segmentation procedure, providing robustness regardless of eventual differences in lightness. Color segmentation was performed using the mean-shift algorithm as Reference [50] (Supplementary File 3). Morphological erosion [51] was employed to remove pixels on plant borders, avoiding readings that could mix plant and background temperatures. Segmented canopies and masks produced after morphological erosion are shown (Supplementary File 4).

The Flir T-420 thermal camera uses two different sensors to generate thermal and visible images. Therefore, such images do not present the same resolution and they are not aligned (Supplementary File 5A,B). Thus, we align them by using a projective transform estimated by the least-squares method and a set of corresponding points [52]. A human annotator identified the corresponding points (Supplementary File 5C), which were used for the proper alignment of visible (Supplementary File 5D) and thermal images (Supplementary File 5E). All the procedures were implemented as a Python computer program. The NumPy package was employed for array manipulation. Mean-shift implementation was provided by the Scikit-learn package [53]. Color space conversion, morphological operations, and projective transform estimation were implemented by the Scikit-image package.

#### 4.2.7. Aboveground Biomass and Grain Yield

The maize plants were harvested at the end of the cycle, when all the leaves were already completely senescent, that is, with the characteristic yellowish color. First, the ears were harvested and, then, the remainder of the plant aerial parts, including culms and leaves. Freshly harvested aerial parts and grains were forced air oven dried at 70 °C/72 h and 105 °C/24 h, respectively. Both aerial parts and grain yield were expressed on a dry matter basis.

### 4.3. Experimental Design and Statistical Analysis

A completely randomized design was used to assign two treatments (control x drought) in four maize genotypes (BRS 1030, BRS 1010, 2B 707 and DKB 390) and five replicates. The data of all variables, which included those relative to gas exchange (*A*, *gs*, *E*, *Ci*, *iWUE*), photochemical (*Fo*, *Fm*, *Fv*/*Fm*), leaves (total, wilted, dead), soil water content (SWC), daily water consumption (DWC), leaf relative water content (LRWC), canopy temperature (CT), aboveground biomass (AGB), and grain yield (GY), were tested for homogeneity of variance and normality of residuals by Cochran’s Q and Shapiro–Wilk tests, respectively. Analyzing each treatment alone (control or stress), but not together, data of all the variables showed homogeneity of variance and normally distributed residuals. Analyzing the variables within each factor, there was homogeneity of variance for DWC and AGB (treatment), *A*, *gs*, *E*, LRWC, DWC, SWC, AGB, and CT (genotype) and DWC, AGB, GY, and CT, phtochemical and leaf variables (interaction). When the factors (treatments and genotypes) or their interactions were significant, the means were compared by Tukey’s test (*p* ≤ 0.05). All the statistical analyses were performed by using the statistical program STATISTICA version 12 (www.statsift.com, Tulsa, OK, USA). Lastly, we correlate the most important variables to each other. Pearson’s correlation was used to test the strength and direction of linear relationships between pairs of continuous variables. Pearson’s correlation coefficient (*r*) was interpreted according to [54]. Thus, *r* equal to 0.00–0.30 means negligible correlation, while 0.30–0.50, 0.50–0.70, 0.70–0.90, and 0.90–1.00 means, respectively, a low, moderate, high, and very high uphill or positive linear relationship. The interpretation for these same values of *r*, but with negative sign, follows the same logic. The difference is that the linear correlation is negative or downhill.

## 5. Conclusions

Thermography was able to discriminate the most contrasting maize genotypes for drought tolerance in a similar way to the field phenotyping in a series of previous experiments in the target environment, in which only grain yield has been considered as a criterion. Although there was a negative correlation between grain yield and plant canopy temperature, it does not necessarily mean that plants whose canopies were maintained cooler under drought achieved the highest yield. Depending on the genotype, drought causes changes in plant leaves and canopy structure, which can alter the absorption and dissipation of energy. Such changes can affect yield and canopy temperature to different degrees. Therefore, further studies should be addressed for a broader understanding of the relationship between canopy temperature and grain yield in maize. In this context, the study of the variables expressed under drought, which influence the canopy temperature, more related to yield (e.g., gas exchange) or survival (e.g., wilt and rolling), are of fundamental importance.

## Supplementary Materials

Supplementary materials can be found at https://www.mdpi.com/1422-0067/20/9/2273/s1.

## Abbreviations

*A*	Net CO_2_ Assimilation Rate
AGB	Aboveground Biomass
AGL	Aboveground Level
BRS	An acronym that identifies materials from the breeding program led by Embrapa
CCI	Chlorophyll Content Index
*Ci*	Intercellular CO_2_ concentration
CNPMS	The National Maize and Sorghum Research Center
CT	Canopy Temperature
DKB	An acronym that identifies materials from the breeding program led by DEKALB
DWC	Daily Water Consumption
*E*	Transpiration rate
Embrapa	Brazilian Agricultural Research Corporation
F_m_	Maximum quantum yield of dark-adapted leaf
F_0_	Minimum quantum yield of dark-adapted leaf
F_v_/F_m_	Maximum quantum yield of photosystem II
*gs*	Stomatal conductance to water vapor
GY	Grain Yield
*i*WUE	Intrinsic Water Use Efficiency
LRWC	Leaf Relative Water Content
PSII	Photosystem II
RGB	Red, Green and Blue color model
SWC	Soil Water Content
UAV	Unmanned Aerial Vehicle
