# Supplementary material for: Using Thermography to Confirm Genotypic Variation for Drought Response in Maize
Source: Int J Mol Sci. 2019 May 8;20(9):2273. doi: 10.3390/ijms20092273 (PMC6540190; doi:10.3390/ijms20092273)
Supplement: Supplementary file 1 [file ijms-20-02273-s001.pdf]

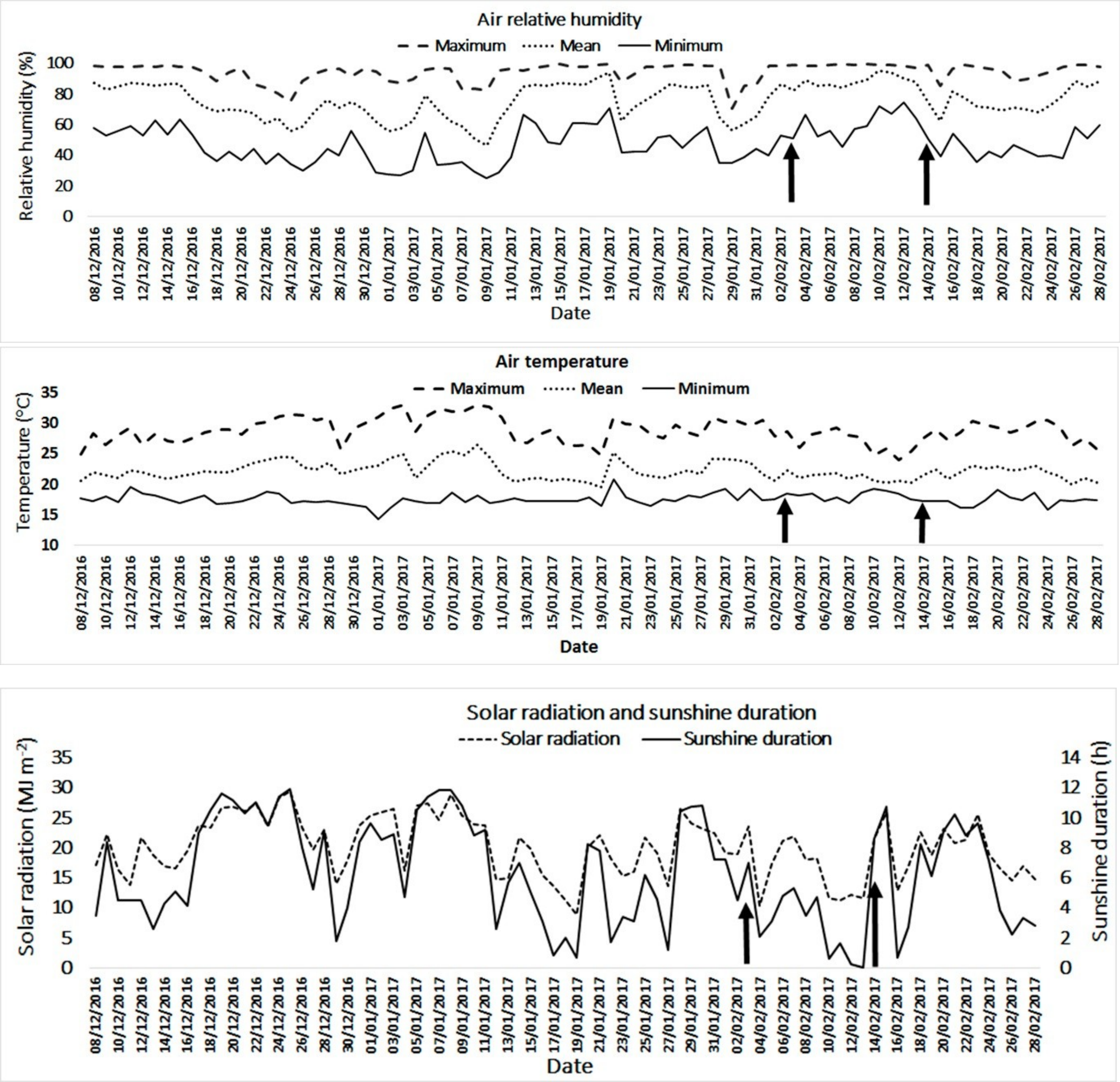

Supplementary File 1. Daily weather data collected from a weather station close to the experimental site. The two arrows indicate the beginning (03/02/2017) and the end (14/02/2017) of drought.

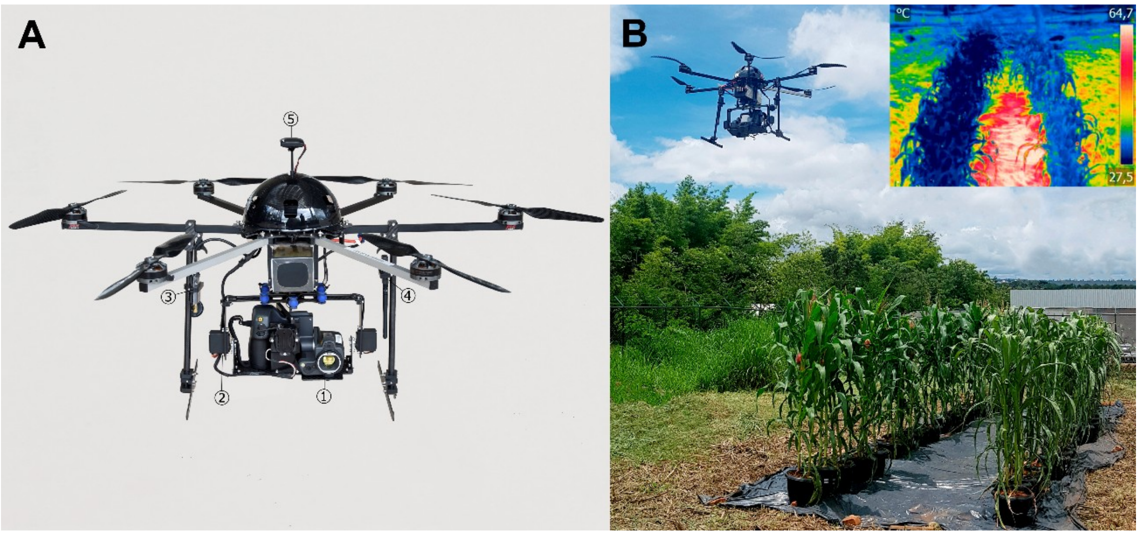

Supplementary File 2. Laboratory-built solution for taking aerial images. (A) Unmanned aerial vehicle carrying the thermal imager used for imaging of maize plants; 1-thermal imager; 2-gimbal; 3-video transmitter; 4-flight data transmitter; 5-GPS and compass. (B) UAV overflight of the experimental area for visible and thermal images record. In this panel are inserted an aerial thermal image (above) and a side visible image (below).

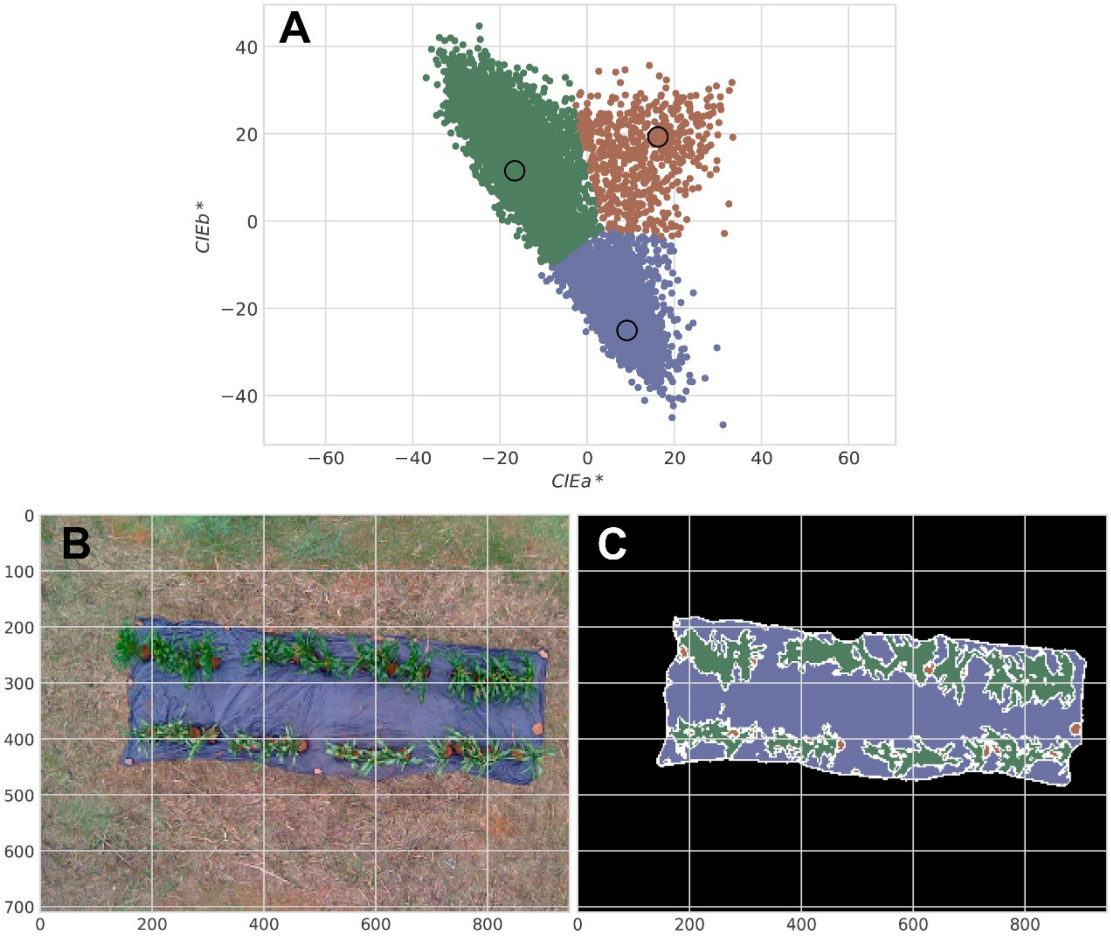

Supplementary File 3. Color segmentation applied for identification of plant pixels. (A) Color segmentation in the a*xb* space by mean-shift clustering. (B) Original RGB image. (C) Segmented image presenting three pixel classes – the classes can easily be associated to leaves, plastic background and soil/rocks. *Images were captured at an UAV flight height of 20 m AGL*.

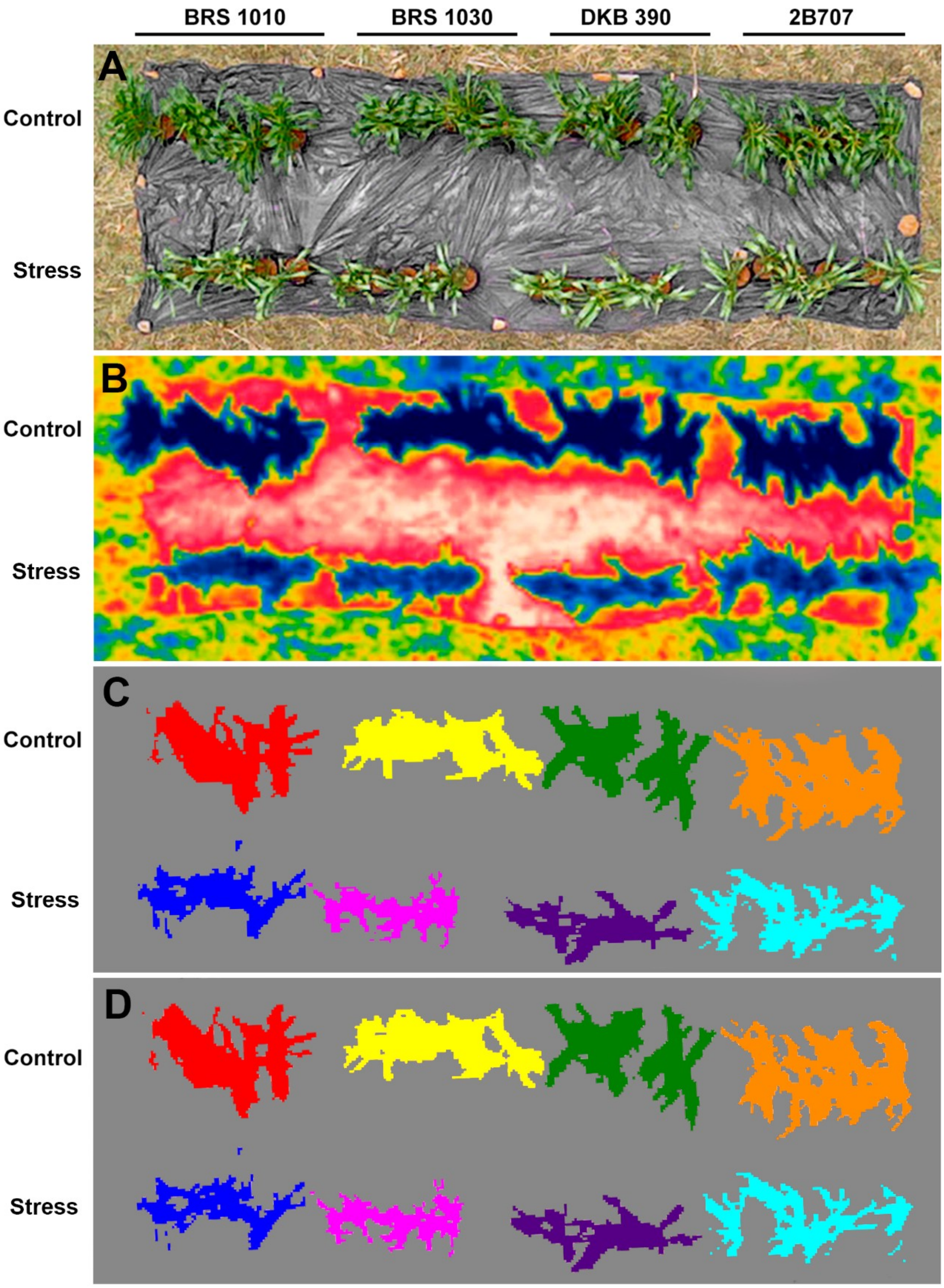

Supplementary File 4. Image segmentation process for plant pixels segregation. (A) Original visible and (B) thermal image – treatments and genotypes are indicated on the figure. (C) Masks produced after color-based segmentation. (D) Final masks, produced

after morphological erosion to exclude borderline pixels that could mix canopy and background signals. *Images were captured at an UAV flight height of 20 m AGL.*

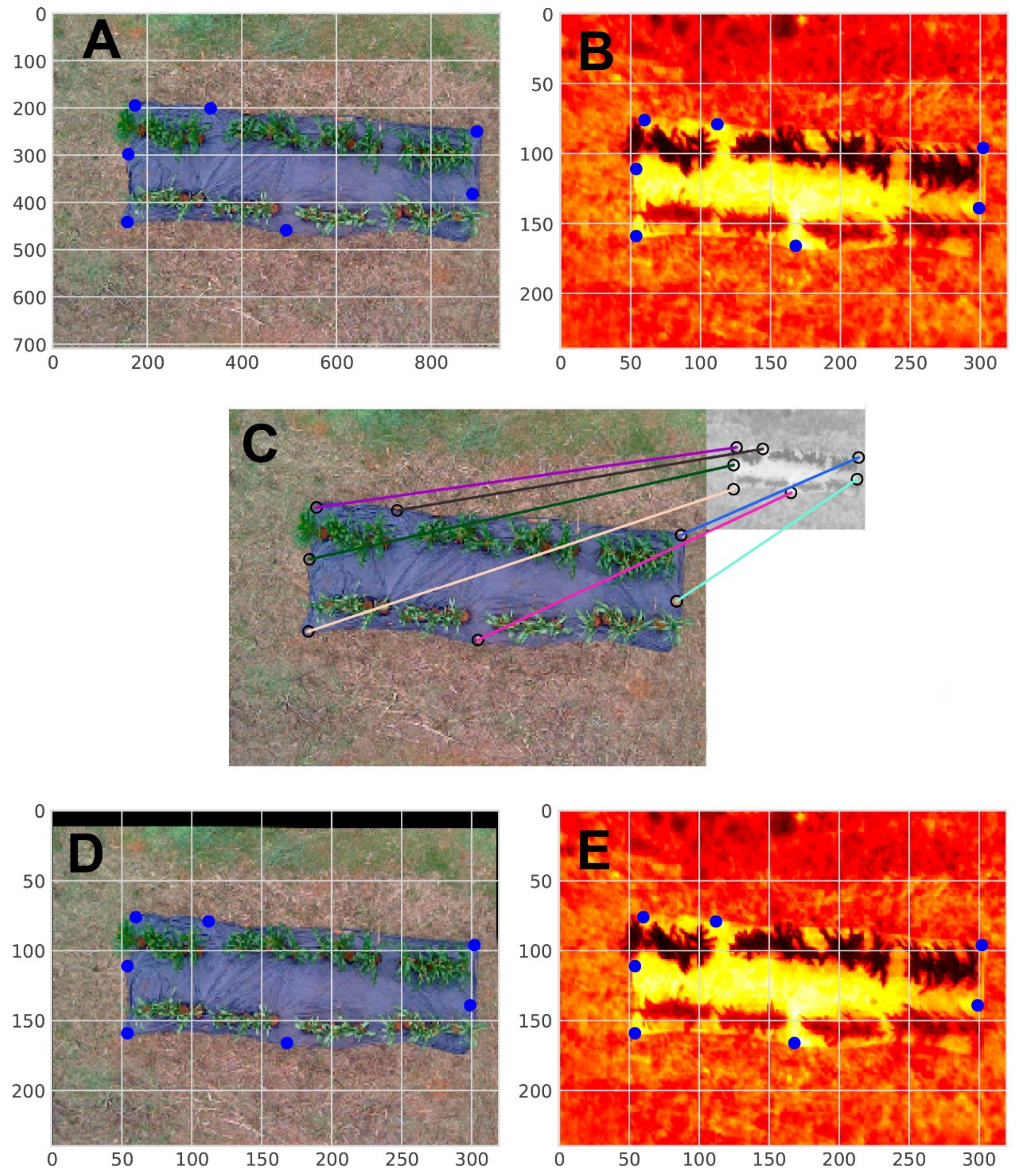

Supplementary File 5. Visible and thermal images alignment process. (A) 947x710 visible and (B) 320x240 thermal image showing the same experimental area. The blue dots show corresponding markers in both images. (C) Correspondent points used to estimate a projective transform for images alignment. (D) Transformed (warped) visible and (E) thermal image properly aligned. *Images were captured at an UAV flight height of 20 m AGL.*
